# Icteric Urine

**Published:** 1878-11

**Authors:** 


Icteric Urine.—When jaundice is not well marked, a great assistance is derivable from examination of the urine. The following three tests are employed by Prof. Hardy before his clinical class with marked success: 1. *Chloroform:* When this is poured upon normal urine, it sinks by reason of its great density to the bottom of the test-glass, exhibiting there a crystalline transparency. If we pour it on the icteric urine, and, having shaken the test-tube plugged by the thumb, leave it quiet for a moment, the chloroform deposit contrasts strongly by its dull color with the yellow of the superficial layers—the yellow color being deeper in proportion to the quantity of bile in the urine. It is an excellent test of icteric urine. 2. *Iodine:* When the iodine is poured upon the icteric urine the mixture must not be shaken. At the upper part of the tube three very distinct colors are observable—the first layer formed by the tincture is violet; below this is a kind of diaphragm of a sea-green color; and the third layer, consisting of the urine, and occupying the lowest part, is yellow. 3. *Nitric Acid:* When this agent has been poured in, the mixture, after shaking, assumes a bottle-green color, passing into an olive. This is an entirely special and very characteristic appearance.—*Med. Times and Gaz.*, Sept. 21, 1878, from *Rev. de Therap.*, Aug. 15.

